# Exploring Cooperative Transboundary River Management Strategies for the Eastern Nile Basin

**DOI:** 10.1029/2017WR022149

**Published:** 2018-11-23

**Authors:** Kevin G. Wheeler, Jim W. Hall, Gamal M. Abdo, Simon J. Dadson, Joseph R. Kasprzyk, Rebecca Smith, Edith A. Zagona

**Affiliations:** ^1^ Environmental Change Institute University of Oxford Oxford UK; ^2^ Water Research Center University of Khartoum Khartoum Sudan; ^3^ School of Geography and Environment University of Oxford Oxford UK; ^4^ Department of Civil, Environmental and Architectural Engineering University of Colorado Boulder Boulder CO USA; ^5^ U.S. Bureau of Reclamation Washington DC USA

**Keywords:** modeling, Nile, Renaissance Dam, negotiation, policy, cooperation

## Abstract

A water resource modeling process is demonstrated to support multistakeholder negotiations over transboundary management of the Nile River. This process addresses the challenge of identifying management options of new hydraulic infrastructure that potentially affects downstream coriparian nations and how the management of existing infrastructure can be adapted. The method includes an exploration of potential management decisions using a multiobjective evolutionary algorithm, intertwined with an iterative process of formulating cooperative strategies to overcome technical and political barriers faced in a transboundary negotiation. The case study is the addition of the Grand Ethiopian Renaissance Dam (GERD) and considers how its operation may be coordinated with adaptations to the operations of Egypt's High Aswan Dam. The results demonstrate that a lack of coordination is likely to be harmful to downstream riparians and suggest that adaptations to infrastructure in Sudan and Egypt can reduce risks to water supplies and energy generation. Although risks can be substantially reduced by agreed releases from the GERD and basic adaptations to the High Aswan Dam, these measures are still insufficient to assure that no additional risk is assumed by Egypt. The method then demonstrates how improvements to water security for both downstream riparians can be achieved through dynamic adaptation of the operation of the GERD during drought conditions. Finally, the paper demonstrates how the robustness of potential management arrangements can be evaluated considering potential effects of climate change, including increased interannual variability and highly uncertain changes such as increases in the future persistence of droughts.

## Key Points

- A framework is introduced to support transboundary river negotiations regarding new infrastructure operations
- A multiobjective evolutionary algorithm is applied to explore trade‐offs in cooperative operation of the large reservoirs on the Eastern Nile
- The method evaluates the robustness of cooperative reservoir operation strategies to deep uncertainties

## 1 Introduction

The kingdoms and countries that have governed the lower Nile River Basin have long recognized their extraordinary reliance on the abundant rain that falls in distant upstream lands and acknowledged Ethiopia as the primary source of their annual floods (Erlikh, 2002). The furthest downstream nation, Egypt, considers the Nile to be its only significant source of water and the protection and reliability of its flows to be an existential priority. However, coordinated management of water‐related infrastructure across the basin's international borders to secure this need has rarely existed, aside from brief colonial efforts to assure reliable flows for agricultural productivity of the Nile delta (Hurst et al., 1947; Tvedt, 2004).

Following rapidly increasing consumptive uses of the Nile River over the last century and projections of demands expected to significantly exceed the average annual supplies (Nile Basin Initiative [NBI], 2012), the countries have acknowledged the need for joint planning and management. Numerous studies have demonstrated how the river could be jointly developed (Arjoon et al., 2014; Blackmore & Whittington, 2008; Block et al., 2007; Digna et al., 2018; Goor et al., 2010; Guariso & Whittington, 1987; Jeuland & Whittington, 2013; van der Krogt & Ogink, 2013; Wheeler & Setzer, 2012); however, less attention has been given to specific reservoir operations and the process of reaching an agreement for coordination between countries. New infrastructure that could change the flows of the river should be managed with existing infrastructure to improve the reliability of water to all parties while protecting the riverine environment. For agreement to be reached, modification of reservoir management must consider changes in the context of historical operational arrangements and the complexities of negotiating a new paradigm of coordination. Management of the Nile is particularly challenging given that water rights are highly politicized and decisions of exploitation are often guided by ambitious national development plans and complex political dynamics (Cascão, 2008). Although waters of the basin are approaching a state of complete utilization and the risks of noncoordination are well known (Jeuland et al., 2014), the current operations of the major dams and reservoirs remain uncoordinated across international borders.

The imminent completion of the Grand Ethiopian Renaissance Dam (GERD) on the Blue Nile has accelerated the urgency for cooperative management between the countries of Ethiopia, Sudan, and Egypt (Cascão & Nicol, 2016). This hydropower project promises to significantly increase access to electricity within Ethiopia and across eastern Africa; however, concerns have been raised that it will cause increased upstream consumptive uses of the river and may lead to Ethiopia withholding water at critical times, putting Egypt at risk of greater water insecurity (Massachusetts Institute of Technology, 2014; Taye et al., 2016; Whittington et al., 2014). Despite decades of anticipation of Ethiopia's goals to develop the Blue Nile, a chasm still exists between the many technically sound or economically optimal solutions that have been proposed and the political willingness of the various countries to adopt them.

In this research, we propose a systematic and outcome‐oriented process that blends both technical and political elements of multiobjective decision making and then demonstrate this process to consider arrangements for operating the GERD alongside other major infrastructure. The process leverages recent advances in water resource models and optimization algorithms, which are used to propose cooperative mechanisms to inform an active negotiation. The process demonstrates a pathway to move from conceptual notions of cooperation to identifying tangible operational policies that could be considered within an evolving negotiation.

### 1.1 Approaches for Managing Transboundary Water Conflicts

Increasing water stress in many regions of the world has increased the potential for transboundary water disputes (Bakker & Duncan, 2017; UNEP‐DHI and UNEP, 2016). Although direct conflicts over transboundary rivers are historically rare (Wolf, 2007), peaceful agreements may not continue to be the most likely outcome given increasing water demands and climatic uncertainties. Such is the case in the Nile Basin (NBI, 2012; Siam & Eltahir, 2017). To encourage cooperation, there is a need for robust and practical approaches that address both physical and political dimensions of transboundary water management while considering increasing uncertainty in future water stresses.

Computer models have long been used by water resource engineers and economists to facilitate the process of planning and collaborative decision making (Loucks, 1992; Thiessen & Loucks, 1992). Simulation models examine potential management arrangements through scenario analysis, while optimization can demonstrate the benefits of strategic water allocation and multiobjective operation of infrastructure (Wurbs, 1993). Various approaches have been proposed to translate optimized model outputs into reservoir management policies (Anghileri et al., 2013; Herman & Giuliani, 2018; Ngo et al., 2007). However, developing actionable operational guidelines remains a challenge. Alternatively, hydropolicy simulation models are designed to accurately reflect reservoir operations and provide transparency of the data and algorithms used to enhance their legitimacy among policy makers and stakeholders. A defining characteristic of these models is their ability to simulate essentially any management policy that can be envisioned, allowing alternative management rules to be tested and readily implemented into practice (Wheeler et al., 2018).

Multiobjective analysis aims to quantify the synergies and trade‐offs serving a variety of interests (Haimes & Hall, 1974; Vemuri, 1974). In recent years there has been an emphasis on multiobjective evolutionary algorithms (MOEAs) that use search techniques to identify a *nondominated* set of management plans that approximate multidimensional Pareto fronts between competing objectives (Reed et al., 2013). A management decision that is *Pareto optimal* implies that any incremental change to improve one objective comes at the cost of another objective. This analysis is useful to understand how development in one country may or may not conflict with the interests of a coriparian country. MOEAs can be coupled with hydropolicy simulation models to explore a wide variety of potential management approaches that demonstrate varying degrees of transboundary cooperation.

Numerous applications of MOEAs in water resources have emerged in recent years (Ahmadi et al., 2014; Giuliani et al., 2014; Giuliani, Anghileri, et al., 2016a; Herman et al., 2014; Kasprzyk et al., 2013; Mortazavi‐Naeini et al., 2014; Smith et al., 2016; Watson & Kasprzyk, 2017; W. Wu et al., 2016), several of which demonstrate the conflicting objectives of competing stakeholders. Maier et al. (2014) argue that the primary focus of MOEA research has been on improving the algorithms and note the lack of applications to real‐world problems. While applications to address the needs of multiple stakeholders in a river system are increasingly common (Herman et al., 2014; W. Wu et al., 2016), to date there have been few that address the challenges of transboundary rivers. Giuliani, Anghileri, et al. (2016a) apply the Borg MOEA (Hadka & Reed, 2013) to the Red River Basin, including parts of China, Laos, and Vietnam, to assess trade‐offs between hydropower, water supply, and flood damages under current and future hydrologic projections. They identify optimal operations using a Direct Policy Search method (Giuliani et al., 2014) and demonstrate how the use of MOEAs to construct policies can be effective to manage the challenges of dimensionality, nonlinear systems, and multiple objectives (Giuliani, Castelletti, et al., 2016b). Geressu and Harou (2015) apply the ɛ‐NSGAII MOEA algorithm (Kollat & Reed, 2006) to evaluate various combinations of proposed reservoirs along the Blue Nile. Assuming optimal operations using rule curves, they evaluate trade‐offs of storage volume, energy generation, and irrigation deficits to conclude that constructing multiple reservoirs of lower capacity is preferable in terms of overall efficiency. Both studies develop operational rules based on optimization procedures rather than encoding current management policies, and neither describes how the countries might incorporate the analysis into a politically negotiated process to reach coordinated management decisions.

While the use of models can identify potential management plans, it is only one component of a complex and often politically driven decision‐making process. Challenges such as adversarial/congenial relationships, concerns of sovereignty over resources, principles of international water law, and embedded power dynamics (Zeitoun & Warner, 2006) suggest that fully automated modeling solutions are unlikely to be adopted in a transboundary context. Based on the participatory concepts of Integrated Water Resource Planning, Langsdale et al. (2013) suggest how collaborative modeling can support multistakeholder decision making. W. Wu et al. (2016) describe a framework for stakeholder participation within an MOEA modeling process to select sources for a municipal water supply, emphasizing the iterative nature of problem formulation, optimization, and review of solutions to provide feedback for improving the formulation. Castelletti and Soncini‐Sessa (2006) introduce Participatory and Integrated Planning that describes a comprehensive sequence of collecting user input, modeling, joint analysis, and negotiation to encourage stakeholder integration. This process is demonstrated on a single reservoir shared by two countries.

The political complexities of reaching negotiated arrangements over transboundary rivers have led to an increased focus on water diplomacy (Islam & Repella, 2015) that seeks to overcome political stalemates through active dialogues that identify ways to increase the overall *value* derived by each party from a water resource. Developed as a pragmatic and mediation‐based approach, this framework emphasizes the incremental consideration of elements or components that meet the objectives of each party, which can then be assembled into a negotiated *package* solution. Water diplomacy cautions against rigid models that consider only predefined management approaches or those that cannot be readily expanded to test new ideas that emerge during a negotiation process. This is why we argue for the use of highly flexible tools such as hydropolicy models coupled with MOEAs to supplement an evolving water diplomacy process.

Efforts by the Nile Basin Initiative to develop a model‐supported approach to reach agreements over basin‐wide management has produced numerous studies of potential development of the eastern Nile Basin (Barbour et al., 2013; Bates et al., 2013; Hassan, 2012; NBI, 2012; van der Krogt & Ogink, 2013; Wheeler & Setzer, 2012) as well as the development of a decision support system to assimilate and analyze basin data (NBI, 2011, 2014). However, a practical challenge remains to identify how an analytical model can be applied within a politically charged context such as the Nile, particularly where unilateral development of infrastructure by one party potentially affects downstream riparian nations. This is complicated by the fact that the severity of the impacts is a function of the willingness of the parties to coordinate management. Although well suited for this purpose, the combination of a hydropolicy model and MOEA has not yet been placed into this practical context.

### 1.2 New Opportunities for the Nile

The method proposed in this work seeks to address the challenge of transboundary water management by using MOEAs to search for management solutions (Kasprzyk et al., 2013). Following the example of Smith et al. (2016) that integrates an operational hydropolicy model with an MOEA to explore solutions for improved management decisions in a complex municipal system, we extend this approach to a negotiation context, which can augment the exiting participatory frameworks described above (Castelletti & Soncini‐Sessa, 2006; W. Wu et al., 2016). The method is applied to a current situation on the Nile suggesting how it *could* be used to facilitate ongoing analyses that provide support to negotiations over the management of the GERD. However, like other Nile studies, it is hypothetical in nature and does not describe its actual use within the negotiations or an outcome that is yet to be concluded.

Unlike previous Nile studies that use optimization to demonstrate water management solutions that differ significantly from current river management practices and are difficult to interpret into actual operations, this study begins with current detailed reservoir operations and seeks to adapt them to a new reality with the GERD operating as a part of the river system. Through an analysis of possible scenarios, we demonstrate how a modeling framework can be used to explore potential pathways of coordination among the countries of Ethiopia, Sudan, and Egypt. The approach acknowledges the unique and competing perspectives of the countries and then looks for opportunities through which the current management of the river can be adapted alongside the establishment of operational guidelines for the new infrastructure.

Our flexible approach is particularly relevant in a transboundary context where differing views of economic values are common, internal political pressures to resist any compromise exist, and concerns over national sovereignty are pervasive. Such divergent perspectives prevail on the Nile, and hence we adopt a preference approach that assumes a predevelopment condition is initially acceptable or preferred by Egypt and Sudan and management of the GERD for the sole purpose of hydropower production is preferred by Ethiopia (F. Negash, personal communication, 26 July 2013). The method considers all perspectives equally and then proceeds to find incremental cooperative arrangements that can potentially yield mutually acceptable outcomes, including consideration of the principle of *no significant harm* derived from international water law (Salman, 2016; Yihdego & Rieu‐Clarke, 2016). We intentionally do not address the wider issue of water rights, however, due to competing interpretations of historical agreements and obligations. The focus is on assessing the outcomes for various actors under a range of different coordination, development, allocation, and hydrological scenarios, which allows solutions to be tested and refined to reach an outcome that is considered acceptable by the parties involved. We do not attempt to seek an elusive optimum based on prioritized or aggregated objectives, but instead we use optimization to search solutions along the Pareto frontier of the various interests of the different actors. In other words, we emphasize satisficing rather than optimizing behavior among negotiators.

While the method addresses the situation on the Nile regarding the GERD, it is applicable for any proposed water resource development in one riparian country that may have an effect on other coriparian countries. Contemporary examples include the Ilisu Dam on the Tigris River in Turkey, which is opposed by downstream Iraq, and the construction of the Xayaburi Dam on the Lower Mekong River, which has faced opposition from Vietnam and Cambodia. With increasing populations and water demands, developments such as these are increasingly common and thus our insights can be widely applied in many future contexts.

## 2 Nile Basin Context

### 2.1 Basin Description

**Figure 1:**
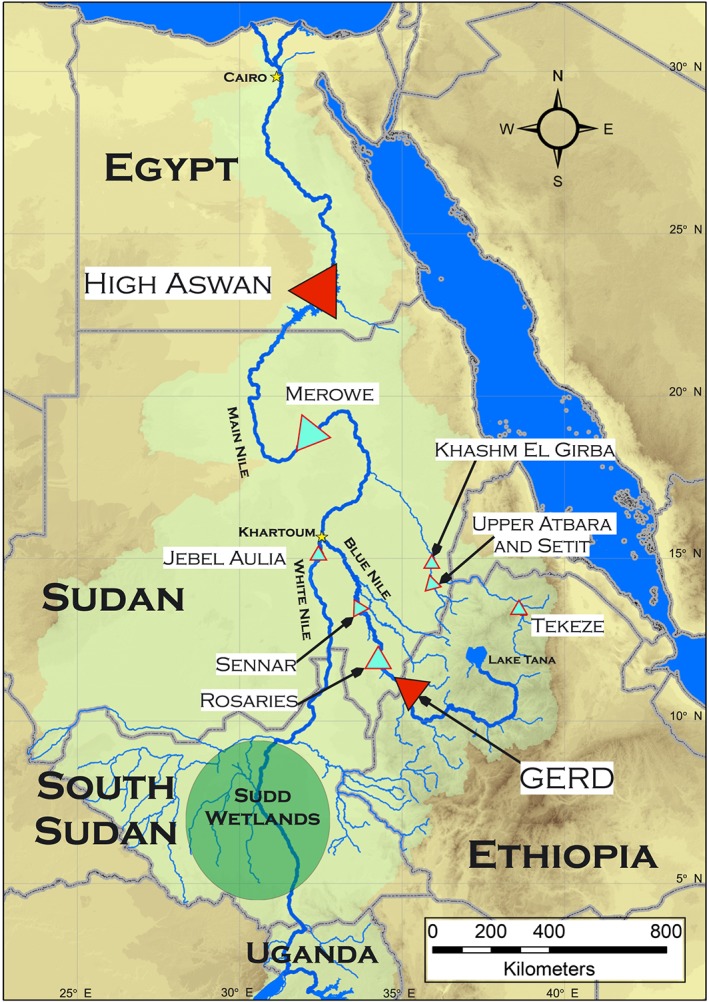
The eastern Nile River Basin with major infrastructure projects.

The Nile Basin includes portions of 11 countries, Burundi, Democratic Republic of Congo, Egypt, Eritrea, Ethiopia, Kenya, Rwanda, South Sudan, Sudan, Tanzania, and Uganda, and spans an area of 3.18 × 10^6^ km^2^. One major branch of the Nile begins in the Equatorial Lakes region and flows from Uganda into the Sudd wetlands of South Sudan and then continues as the White Nile into the Sudanese capital city of Khartoum. The Blue Nile begins in Ethiopia and flows into Sudan where it joins the White Nile in Khartoum. Downstream of this confluence, the combined waters flow as the main Nile from Sudan into Egypt and northward to the Mediterranean Sea. The Sudd wetlands provide a natural hydrologic buffer resulting in relatively steady average monthly flows of the White Nile ranging from 580 to 1,270 m^3^/s at Malakal in South Sudan, while the seasonal fluctuations of the Blue Nile are pronounced with average monthly flows ranging from 150 to 5,600 m^3^/s at the Soba gauge in Khartoum. Given the hydrologically distinct Equatorial Lakes region and buffering effect of the Sudd wetland (Sutcliffe & Parks, 1999; Tate et al., 2001), the eastern Nile Basin is delineated to exclude the area upstream of the Uganda‐South Sudan border and thus encompasses the countries of South Sudan, Sudan, Ethiopia, and Egypt. This area forms the domain of our study to assess cooperative management of the GERD (Figure 1).

The completion of the GERD will be the latest infrastructure in a long history of efforts to engineer the flows of the Nile to meet the needs of a growing population. The Low Aswan Dam (1902) in Egypt and the Sennar Dam (1925) and Jebel Aulia Dam (1937) in Sudan were the first modern attempts to construct barriers across the Main Nile, Blue Nile, and White Nile, respectively. A 1959 agreement between Egypt and Sudan initiated the construction of the Roseries Dam (1966, expanded in 2013) and Khashm El Girba Dam (1964) in Sudan and the High Aswan Dam (HAD; 1970) in Egypt (Nile Treaty, 1959). The Merowe Dam (2009), the Upper Atbara and Setit Dam complex (2016), and the heightening of the Roseries Dam (2013) demonstrate Sudan's modern expansion of water management infrastructure. The Finchaa (1973, expanded in 2012), Tekeze Dam (2009), and the Tana‐Beles hydropower generation station (2010) are Ethiopia's major endeavors to date.

Ethiopia has long considered large hydropower development of the Blue Nile as an important part of its pathway to improve the economic condition of the country (MOFED, 2010; USBR, 1964). The construction of the GERD was announced in 2011, and Ethiopian leaders have often stated that it will not cause significant harm to the downstream countries of Sudan and Egypt (Gebreluel, 2014). While some argue that newly created storage could pose a risk if water is withheld during critical periods (Hamed, 2018), it may also serve as a benefit to these downstream countries in times of extended drought (Whittington et al., 2014). Sudan has recognized the benefits that the dam is likely to provide through sediment reductions, hydropower uplift, flood management, and the potential for increased agricultural development (Basheer et al., 2018). Egypt has been reluctant to support the project, fearing reductions in future water use (Cascão & Nicol, 2016; Tawfik, 2016). At the current time, the countries are negotiating a plan to fill the reservoir, and no solutions have been put forth to coordinate the GERD with the existing infrastructure in Sudan or Egypt, creating a potentially precarious situation.

### 2.2 Previous Nile Cooperation Studies

Numerous studies have been conducted that analyze the cooperative development potential of the Nile. Dating back to the colonial era, proposals were considered to expand storage capacity in the two major tributaries to provide more reliable flows for irrigation in Egypt (Hurst et al., 1947). This need was largely met when Egypt constructed the HAD, which effectively postponed the need for cooperative decision making with upstream nations yet at the expense of substantial evaporation losses from Lake Nasser.

Analyses of cooperative arrangements have continued throughout the decades. Using a classical optimization approach, Guariso and Whittington (1987) developed a multiobjective model to maximize future Ethiopian hydropower production alongside agricultural production in Sudan and Egypt. They demonstrated that upstream hydropower development was compatible with, and even beneficial to, downstream agricultural production in Sudan and to a lesser extent in Egypt. The economic value of basin‐wide cooperation was further demonstrated by Whittington et al. (2005) using a deterministic Nile Economic Optimization Model (NEOM). The model was used to evaluate the balance between the economic benefits of hydropower production and irrigation water withdrawals while considering the economic pressures of evaporation and seepage losses. The study concluded with annual economic benefits of $7–11 billion, neglecting the costs of new infrastructure. X. Wu and Whittington (2006) coupled the NEOM with a game theory approach to compare the economic benefits of various coalitions with noncooperative developments. More recently, Jeuland et al. (2017) reapplied the NEOM in the context of the GERD to examine the economic benefits of system‐wide optimization compared to forms of constrained noncooperative development including varied adherence to the legal principle of no significant harm and the 1959 treaty between Egypt and Sudan. Each of these models used a classic optimization approach that assigns releases to maximize a collection of benefits yet with different objectives, constraints, and aggregations of actors acting in an optimal way.

Block and Strzepek (2010) applied stochastically generated hydrologic scenarios influenced by potential effects of climate change to conduct a risk‐based analysis of the cost‐benefit ratios of multiple developments in Ethiopia. This study addressed the critical aspects of reservoir filling and staggering of multiple construction projects and considered two flow retention policies for the future Ethiopian reservoirs. While demonstrating economic advantages of Ethiopian development, they highlighted the significant need for consideration of the temporal aspect of construction. Furthermore, this study highlighted the decreased benefit‐cost ratio of development when doubling the frequency of El Niño events to represent greater hydrologic variability. While downstream flow requirements into Sudan and coordination among potential Ethiopian Reservoirs systems were inherent in their systems optimization approach, Sudanese and Egyptian reservoirs were not included in their analysis and thus coordinated operation between the countries was not considered. Goor et al. (2010) developed a stochastic dual dynamic programming model for the eastern Nile Basin and used 30 synthetically generated hydrologic scenarios to demonstrate the benefits of cooperative operations potential development in the Blue Nile with downstream reservoirs. Arjoon et al. (2014) reapplied this framework specifically considering the GERD, showing strong economic benefits of cooperation. Similar to the studies mentioned previously, this framework assumes optimized reservoir management across the international borders, which effectively replaces existing management with an ideally coordinated system. While this approach provides a view of the idealized benefits of coordination, the current reservoir operational rules are not replicated and it neglects the difficult negotiation process to achieve their ideal situation.

Similar to Block and Strzepek (2010), Jeuland and Whittington (2014) examine a wide variety of Blue Nile development scenarios under different deep uncertainties including multiple multireservoir configurations, sequencing and timing of their construction, sizing of the reservoirs, and operating rules. They use a thorough simulation approach rather than an optimization approach, however, to consider a variety options and uncertainties. Choices regarding dam operating rules are presented in a simplified binary choice of *hydropower based* versus *downstream coordination* within their hydrological routing model. While this limits its applicability to simulate the wide variety of reservoir operations that could be envisaged throughout the course of a negotiation, this work introduces one explicit coordination strategy suggesting a minimum release from Ethiopian reservoirs if the storage of the HAD falls below 60 billion cubic meters (BCM).

More recent efforts focus specifically on the filling process of GERD. King and Block (2014) and Zhang et al. (2015) consider various GERD reservoir‐filling strategies and evaluate the resulting impacts on the Gezira irrigation diversion in Sudan and inflows into Egypt. These studies highlighted the sensitivity of the strategy chosen and assumptions of climate on the filling process but modeled the system in a simplified way with only the operations of the GERD considered. Finally, Wheeler et al. (2016) introduce the Eastern Nile RiverWare Model (ENRM) to evaluate potential GERD filling policies that reference pool elevations of the HAD.

We note that many previous studies have provided valuable insight into the benefits of cooperation through optimized system operations, yet translating optimized results into operational policies that specify dam operations is notoriously challenging (Yeh, 1985). We also recognize that new methods to derive reservoir operation policies such as Direct Policy Search hold the potential for overhauling system operations (Giuliani et al., 2014). However, the focus of our work is substantially different, as it focuses on how to move from the status quo to mutually preferable management practices. This is particularly salient when the institutions that manage water are highly fragmented and existing practices are resistant to change. In this work, we evaluate the potential upstream‐downstream coordination policies of reservoirs after the GERD filling is complete and describe a model‐supported negotiation process by which that decision‐making process might take place.

### 2.3 Droughts and Climate Changes on the Nile

Records have been kept since ancient times on the flooding and droughts of the Nile (Hassan, 2007) and have been a topic of scientific study for well over a century (Waite, 1904). In north Africa, droughts are directly linked to the economic well‐being of the countries (Block & Rajagopalan, 2007; Strzepek & McCluskey, 2007), and thus numerous scientific endeavors have been made to understand and predict their frequency and severity (Block & Strzepek, 2010; Conway & Hulme, 1993; Conway & Hulme, 1996; Eltahir & Wang, 1999; Siam & Eltahir, 2015).

The tendency for flows to remain above and below average annual volumes over sequential years was noticed during early studies to determine the storage required to control the flows of the Nile (Hurst, 1951). The ratio of the required storage (*R*) to the standard deviation of annual flow (*σ*) were shown to have similar nondimensional values that increased with record length (*N*).

$$\frac{R}{\sigma }={\left(\frac{N}{2}\right)}^{K}$$

Mathematically formalized by Mandelbrot and Wallis (1968), the exponent *K* has become known as the Hurst exponent or Hurst coefficient, a metric of the *persistence* of flows ranging from 0.5 to 1.0 (Sutcliffe et al., 2016). Connections between El Niño–Southern Oscillation signals and the occurrence of flooding and drought conditions have been made (Zaroug et al., 2014), and wavelet analyses have sought to understand the cyclical nature of such events and their implications for water management (Elsanabary & Gan, 2014; Melesse et al., 2010; Zhang et al., 2016). Koutsoyiannis (2003) suggest that multiple‐scale variability of a time series can explain the Hurst phenomenon.

Projecting the effects of global climate changes in the Nile Basin is an evolving area of research with significant implications with respect to impacts and economic investments. According to studies using general circulation models (GCMs), temperatures are generally expected to increase across the basin. Changes in the direction and magnitude of precipitation are far less certain (Beyene et al., 2010; Conway & Hulme, 1996; Di Baldassarre et al., 2011; Yates & Strzepek, 1998), and as a result, projections of runoff and water availability vary significantly. Studies evaluating the climate impacts on the Eastern Nile development potential often focus on analyzing the effects of deviations to potential changes in flows (Jeuland, 2010; Jeuland & Whittington, 2014) or increases in the frequencies of El Niño and La Niña effects (Block & Strzepek, 2010). Combining observational evidence and GCM projections, Siam and Eltahir (2017) demonstrated that hydrologic variability of flows in the Nile has been increasing and is expected to continue to do so into the future due to increased frequency of El Niño and La Niña events, thus increasing the risk of flooding and extended droughts. Their study estimates an increase to the standard deviation of interannual flows to be 50% (±35%) between the 20th and 21st centuries.

While the evidence of increased frequency of these extreme events indeed is alarming, the risk of droughts occurring in sequential years is also a concern. Siam and Eltahir (2017) noted an increased Hurst coefficient in over half of their bias‐corrected GCM projections, and the assessment by Hurst (1951) emphasized that greater hydrologic persistence would require greater basin storage. Although the current state of science provides only a limited understanding of the mechanisms and likelihood of increased durations of droughts, the ability to test whether there is sufficient storage volume in the Nile system to maintain a reliable supply to Egypt is an acute challenge. Mitigating potential impacts through costly infrastructure investments is one approach to managing climate risks (Jeuland et al., 2017); however, improved operation of existing reservoirs (Goor et al., 2010) and reaching transboundary agreements to cooperatively manage infrastructure in the Nile Basin is another approach with longer‐term mutual benefits (Sadoff & Grey, 2002; Tilmant & Kinzelbach, 2012; Whittington et al., 2005).

## 3 Methodological Framework

**Figure 2:**
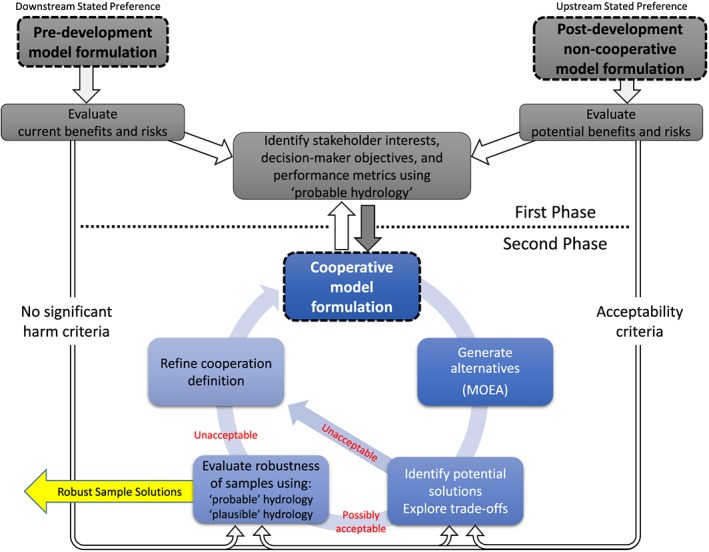
Framework to use models for supporting negotiations when seeking agreements among coriparian nations over new developments in transboundary rivers. MOEA = multiobjective evolutionary algorithm.

In Figure 2, we present a model‐supported process for seeking cooperative water management strategies for new developments in transboundary rivers. The framework addresses the scenario of a proposed new infrastructure development in an upstream riparian country that is likely to affect flows in a downstream country.

Central to the arrangement proposed in Figure 2 is the concept of a *formulation* that consists of four components including (1) a model of the physical configuration of the system with the current or future components represented that significantly affect the management of water, (2) a set of decision rules that encodes how each of the reservoirs and other hydraulic infrastructures are operated and thus defines how water is managed throughout the system, (3) a specific set of objectives that the management of the system seeks to achieve (e.g., avoid shortages and maximize hydropower generation), and (4) a set of selected variables that can be adapted to achieve those objectives.

Using the terminology described earlier by W. Wu et al. (2016) of participants in a real‐world model‐supported process, a formulation is recognizable to domain experts in the basin and can be unambiguously encoded in a simulation model by analysts. In theory, the decision makers (DMs) are national‐level entities or individuals that in principle represent the interests of multiple stakeholders within their geographic domain. With input from the various participants, a variety of formulations can be developed by the analysts that depict how the new and existing infrastructure can be managed within the river system.

### 3.1 First Phase—Exploration of Baseline Conditions

The process presented in Figure 2 begins with an initial phase that focuses on identifying the interests of the stakeholders as the desired objectives, distilling evaluation metrics that quantify these objectives, and then identifying criteria that need to be satisfied for an outcome to be considered acceptable. Objectives may, for example, relate to hydropower production, the reliability of water availability for use, flood control, and sediment control.

Within the first phase, the predevelopment formulation represents the status quo without additional infrastructure development, while a postdevelopment formulation represents a scenario after a proposed change has been made to the system. In a transboundary river scenario, DMs from different countries may take opposing initial positions on how to operate infrastructure or whether the new infrastructure should be constructed at all. A downstream country that is not party to new upstream infrastructure may prefer the benefits, and risks are characterized relative to status quo (i.e., analyzing deviations from current benefits and risks), while the upstream country in which the new infrastructure would be located may claim absolute sovereignty and control of its operation and would prefer to manage it to maximize its own objectives. In this noncooperative case, the upstream country would consider this postdevelopment formulation as the basis for the benefits and risks to be evaluated (i.e., analyzing deviations from potential benefits and risks). These alternative perspectives can be considered a form of *rival framings* (Quinn et al., 2017), and while each may reflect valid national interests, the extent of their compatibility or conflict is uncertain without further analysis.

Using the transboundary scenario described above, this phase exposes the preference structure of the DMs (i.e., the predevelopment formulation and the noncooperative postdevelopment formulation, alongside the objectives of each country) (Raiffa et al., 2002). The results are quantified in modeled outputs that provide evaluation metrics of the objectives as defined by the DMs, established using predefined stakeholder interests. Although this study suggests that acceptability criteria can be derived from models, these criteria can also be defined based on stakeholder consultations (Glicken, 2000), legal principles (Salman, 2007), or concepts such as tolerable risk (Hall & Borgomeo, 2013) if they can be precisely defined. Furthermore, these criteria may also be adaptable throughout the negotiation process.

In this first phase, the system performance is tested across a wide range of hydrological conditions that are consistent with today's climate, using stochastic simulation of streamflow calibrated using observed flow series. The use of synthetic hydrologic sequences avoids any misconception that the baseline is defined by past observed outcomes, allowing counterfactual hydrologies to be considered that could plausibly have materialized in the recent past. This first set of hydrological conditions are considered *probable* and are the basis for the initial negotiations. Stakeholders in the system will be particularly interested in system performance when subjected to observed hydrological conditions, for example, well‐known droughts. Illustrating how new arrangements respond to past conditions can be a powerful way of communicating. Nonetheless, the limitations of using selected observed series should also be emphasized, recognizing that large numbers of stochastic simulations are needed to generate stable risk estimates for different stakeholders.

### 3.2 Second Phase—Exploration of Cooperative Formulations

In the second phase, iterative testing of cooperative formulations explores arrangements in which the new infrastructure would be operated in some manner that considers the needs of coriparian stakeholders. In a river system with established management practices, the operation of new infrastructure can be coordinated with adaptations to existing infrastructure in the basin.

Multiple formulations are structured according to a small set of possible principles for cooperation which reflect increasing degrees of coordination among parties (Sadoff & Grey, 2005) and the component‐based approach described in water diplomacy. A *basic cooperation* formulation assumes there is a commitment of regular releases from a reservoir controlled by an upstream country (i.e., GERD) to a meet the fixed needs of a downstream country (i.e., Egypt). A *continuous cooperation* formulation adds a component of drought protection by assuming releases from the upstream reservoir are adapted based on the hydrologic conditions within the downstream country. Similar coordinated flood planning measures could be envisioned or even more advanced forms of cooperation such as water accounting and transfers between states to increase benefits and minimize harm. The decision to add such cooperative components to an agreement is inherently political yet can be informed by hydropolicy models capable of simulating such elements.

Each cooperative formulation is parameterized according to the set of variables in the formulation that defines how the system would be operated (e.g., reservoir releases and trigger levels). For a given formulation, an MOEA is used to generate a set of parameterizations, each one of which represents a possible solution to the negotiation. This is somewhat different from previous MOEA applications that use less structured formulations that consider combinations of many state variables to derive a policy, which in principle might lead to a somewhat improved global optimum, but the formulation is unlikely to be recognizable or even intelligible to DMs who are familiar with the existing operating rules for the basins. Recognizable cooperative principles provide a tangible basis for negotiation, which can then harness the power of MOEAs to explore the trade‐offs and opportunities implicit in those principles.

The parameterizations explored by an MOEA for a particular formulation result in a set of solutions that must meet minimum acceptability criteria by all parties and balance or optimize the remaining objectives and risks through the negotiation process. To evaluate the benefits and risks of cooperation, the second phase of the analysis looks not only at the range of hydrological variability that is implied by the observed record but also plausible ways in which hydrology may change in the future, in particular due to the effects of climatic change. Therefore, within this second phase, a two‐step exploratory analysis first seeks to identify potential cooperative solutions based on likely or probable conditions as described in the first phase and then explores the extent to which these solutions can withstand possible future changes or, on the other hand, whether there are possible future changes that may threaten the outcomes of cooperation. We particularly focus upon possible future conditions that may be critical to system performance, for example, changes in hydrologic variability or the persistence of prolonged dry spells.

In the first step of the second phase, cooperative formulations are initially evaluated under the probable hydrologic conditions as described earlier in the first phase. Solutions are evaluated by the criteria proposed by the DMs, represented by the no significant harm and *acceptability* criteria in Figure 2. If cooperative solutions cannot be identified that meet the acceptability criteria of all stakeholders under these probable hydrologic conditions, changes must be made to the problem formulation itself through the introduction of new management principles or infrastructure, as shown by the diagonal arrow in Figure 2. Increasing, decreasing, or otherwise altering the methods of cooperation can be considered, such as adding components to the negotiation such as transboundary drought management and flood protection. Alternatively, the acceptability criteria can be reconsidered. If cooperative solutions exist that can meet all assumed stakeholder criteria under probable hydrologic conditions, they are considered possibly acceptable in Figure 2 yet may still be inadequate in future conditions.

In the second step of the second phase, our framework considers *plausible* ways in which the hydrology may change, for example, due to changes in interannual variability or the persistence of prolonged dry spells. Samples are selected from the possibly acceptable solutions to consider their robustness under a wider range of uncertainties or across many plausible states of the world (Hall et al., 2012). These can include expanded hydrologic conditions or variations of any other parameter whose value is unknown, including aspects that are considered *deeply uncertain* or lacking any statistically quantifiable rationale. One example is how a solution responds to a prolonged drought event that is significantly beyond those observable in historical records.

In the MOEA optimization process, we use a combination of objectives that may (i) seek to maximize an objective function (e.g., total hydropower energy production) or alternatively may (ii) seek to maximize the probability of achieving acceptable or satisfactory outcomes across a wide range of possible states of the world (Hall & Borgomeo, 2013; Herman et al., 2015). The latter approach may be thought of as being a version of *robust satisficing* in which the DM seeks to satisfy minimum performance requirements under the widest range of future conditions (Ben‐Haim, 2006). Herman et al. (2015) describe a practical approach to measuring robustness as calculating the fraction of scenarios in which the solution satisfies one or more of the performance thresholds. Decisions can also be sought by considering a wider range of uncertainties initially and the cost of doing so evaluated ex post facto (Giuliani, Anghileri, et al., 2016a; Watson & Kasprzyk, 2017).

Although the process described above identifies and uses stakeholder‐driven criteria as acceptability thresholds, we acknowledge that an acceptable solution is unlikely to be a purely mathematical decision and political factors are likely to have significant influence on the outcome in a transboundary context. A negotiation, however, can be informed by identifying scientifically sound and nondominated alternatives, filtering out combinations of decision variables that demonstrate politically unacceptable outcomes, and iteratively reformulating the problem as needed to meet the dynamic nature of negotiations. As highlighted in the water diplomacy literature, the primary question to be asked is whether all parties can live with the proposed solution (Islam & Susskind, 2012). If this is not the case, changes can be made to the problem formulation itself or the satisficing criteria can be adjusted.

## 4 Application of Methodology

This study applies the model‐supported transboundary negotiation framework to identify water management strategies that the countries may choose to follow. We analyze potential operations of the GERD and the HAD while considering the risks of increased hydrological persistence as an example of testing robustness to climatic uncertainties. The ENRM (Wheeler et al., 2016) is coupled with the Borg MOEA (Hadka & Reed, 2013) to explore the ability of the reservoirs to meet the objectives of all three countries. Following Smith et al. (2016), the combination of the RiverWare hydropolicy modeling platform (Zagona et al., 2001) with the Borg MOEA provides a powerful process to search for management strategies in a complex decision space. Borg has been shown to effectively handle complex, nonlinear, nonconcave problems with superior performance efficiency (Hadka & Reed, 2012) when searching for nondominated solutions (Giuliani, Castelletti, et al., 2016b; Herman et al., 2014). Potential cooperative arrangements are sought to maximize the individual and collective benefits of the GERD while adhering to the principle of no significant harm relative to a condition prior to the GERD.

### 4.1 Simulation

The ENRM simulation model was configured to study long‐term management strategies after the GERD has completed the filling process. The monthly model operates from 2018 until arbitrary ending date of 2060 and includes 162 inflow nodes and 19 water demands representing major or aggregate water diversions. In addition to the management of the GERD, the model simulates reservoir operations of the HAD and Merowe dams on the Main Nile; Lake Tana, Tana‐Beles hydropower diversion, Finchaa, Sennar, and Roseries dams in the Blue Nile subbasin; the Jebel Aulia dam on the White Nile; and the Khashm El Girba, Upper Atbara and Setit complex, and Tekeze dams in the Tekeze‐Setit‐Atbara subbasin.

A reconstruction of naturalized hydrologic conditions for the eastern Nile Basin from 1900 to 2002 (van der Krogt & Ogink, 2013) was used to generate ensembles of 100 stochastic synthetic flow time series across 162 inflow locations using a simulated annealing algorithm (Borgomeo et al., 2015). The first set of 100 synthetic time series matches the statistical properties of the naturalized hydrology including the Hurst coefficient for hydrologic persistence (current conditions) and is considered to reflect the probable hydrologic conditions. Furthermore, two plausible variations were also generated that represent uncertain future climate conditions. The first increases the interannual standard deviation by 15% based on projections between 2018 and 2060 Siam and Eltahir (2017; +15% ias), and the second increases the Hurst coefficient at each inflow location by 20% (+20% Hurst). The purpose of this exercise is not to conduct an extensive analysis of climate change impacts but to demonstrate how the negotiation‐focused method can incorporate the uncertainties of climate. Many other potential variations to the flow series could be tested within the same framework. For each variation, 100 synthetic time series were generated and each is considered plausible hydrologic conditions that are consistent with the stated scenario, as related to the process in Figure 2. The model incorporates average monthly inflows at various headwater and laterally intervening inflows and then aggregates and routes these flows downstream, taking into account various hydraulic processes such as flow variable seepage and evaporation losses, partial month lags, and storage routing algorithms.

**Figure 3:**
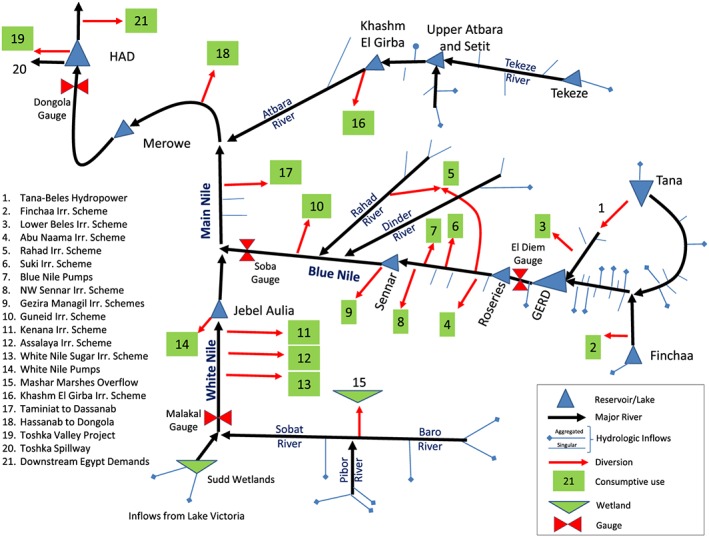
Nile model schematic. Inflows at 162 locations are aggregated for visual clarity.

The consumptive uses are based on projections that have been documented in previous studies. These estimates are not intended to be endorsements of any allocation but necessary modeling assumptions of diversion requests or effectively how much each country might attempt to divert. The HAD is assumed to provide 55.5 BCM/year into the future for use in Egypt, including 4 BCM pumped directly from the Toskha Valley Project (Egypt MWRI, 2005) and the remainder released downstream (Figure 3, items 19 and 20). Annual diversions for Sudanese uses are assumed to total 18.5 BCM throughout this analysis (items 4 to 18). In addition to the Finchaa Irrigation Scheme (item 2), Ethiopia is assumed to increase its consumptive use from the Lower Beles River (item 3) for a total Ethiopian diversion of 2.5 BCM/year (Blackmore & Whittington, 2008), in addition to 1.1 to 2.1 BCM/year diverted from inflows to Lake Tana for irrigation purposes. Evaporative losses from all reservoirs are dynamically modeled in the analysis as additional consumption.

The focus of the method is on adapting existing reservoir operations; therefore, accurately characterizing the current and projected management rules is essential. This allows decision variables to be identified and used in the MOEA optimization. The maximum elevation of the HAD reservoir is 182 m, and the minimum operating level due to hydraulic characteristics of the dam structure is 147 m (Moussa, 2017). The minimum power generation elevation is 159 m, below which the turbines are bypassed to deliver water downstream (D. Amin, personal communication, 29 April 2018). Operations of the HAD include the current drought management plan that reduces releases by 5%, 10%, and 15% when storage volumes fall below 60, 55, and 50 BCM, respectively (Moussa, 2017). Current flood prevention measures require proactive releases to lower the pool elevation below 175 m prior to 1 August, which assures sufficient empty space in the reservoir before the onset of the flood season (Moussa, 2017). Dam operators must also respect a 260 × 10^6^‐m^3^/day maximum release to avoid inundation of the islands downstream in Aswan (Egypt MWRI, 2005).

Modeled operations of the Sudanese reservoirs prior to the GERD reflect the current operational guidelines to meet the variety of objectives specific to each reservoir. The multiobjective reservoirs of the Blue Nile typically operate with a primary purpose of meeting agricultural needs, with seasonal target elevations to allow for sediment transport through low sluice gates. Jebel Aulia operates to meet pumping diversions from the reservoir and hold back flows of the White Nile until the flood of the Blue Nile has passed. While hydropower from the Blue Nile reservoirs is typically a by‐product after agricultural releases are made, the Merowe dam downstream on the Main Nile operates primarily for hydropower but also modifies it operations seasonally to pass sediment and prepare for flood inflows. All reservoirs have minimum outflow requirements that must be met. The reader is directed to Wheeler et al. (2016) for the detailed operations assumptions used in the ENRM. For the current study, the model was updated with increased spatial resolution of demands and recalibrated against historical gage data. The results of this recalibration are in Table 1. According to Moriasi et al. (2007), each result is classified as *good* or *very good*.

**Table 1:** Calibration and Validation Results

Location	Calibration period (1951–1970)			Validation period (1971–1990)		
	Nash‐Sutcliffe	PBIAS	RSR	Nash‐Sutcliffe	PBIAS	RSR
Blue Nile at Kessie	0.99	0.62	0.10	1.00	−1.37	0.04
Blue Nile at El Diem	1.00	−0.41	0.05	1.00	−2.09	0.05
Blue Nile at Khartoum Soba	0.98	0.02	0.13	0.94	−0.62	0.25
Baro at Gambella	0.83	−4.50	0.42	0.93	−1.78	0.26
Sobat at Hillel Doleib	0.85	−1.69	0.39	0.81	−7.03	0.44
Atbara Kilo3	0.89	−1.42	0.33	0.78	−9.97	0.46
White Nile at Malakal	0.89	1.76	0.34	0.85	0.79	0.39
White Nile at Melut	0.89	1.16	0.32	—	—	—
White Nile at Mogren	0.66	−0.97	0.59	0.71	3.96	0.53
Nile at Tamaniat	0.98	0.14	0.14	0.95	0.94	0.23
Nile at Dongola	—	—	—	0.94	−2.61	0.24

*Note*. Metrics include the Nash‐Sutcliffe coefficient, percent bias (PBAIS), and root‐mean‐square error‐observations standard deviation ratio (RSR; Moriasi et al., 2007).

When the GERD is completed, we assume it will be operated primarily for steady hydropower production, with the ability to generate up to 6,450 MW. A minimum operating level of 590 m is assumed, and flood releases are made to maintain a maximum elevation of 640 m. All flows are discharged through the turbines up to the maximum generation capacity, and the remainder are assumed to pass over the spillway. While the contentious process of filling is currently under negotiation, and the result will influence the filling timeline, we explicitly focus our study on operations of the GERD after the filling is complete to understand how the GERD can be managed for mutual benefits over the long term rather than focusing on the impacts of filling. We direct the reader to previous studies that explicitly focus on filling the GERD (King & Block, 2014; Wheeler et al., 2016; Zhang et al., 2015), which is likely to be negotiated in a different time frame compared to possible resolutions over these long‐term coordination decisions.

After the completion of the GERD, operations of the Sudanese reservoirs are assumed to change to meet their primary objectives while making the most of any opportunities that the GERD presents (Basheer et al., 2018). With the vast majority of the sediment captured in the GERD reservoir, the Roseries and Sennar dams will no longer be regularly drawn down to minimum levels to discharge sediment. Our assumption is to maximize the reservoir levels at all times possible, after making releases for Sudanese needs and then spilling the remainder downstream to maintain maximum elevations. Releases are made from Roseries to meet the downstream agricultural needs in Sudan and the minimum flows downstream of Sennar. Flows are passed through turbines whenever possible to allow for energy generation, and regular annual spills are expected to occur to maintain the maximum allowable pool elevation. A similar assumption is made for the Merowe Dam that keeps the elevation as high as possible and makes regular releases to meet energy demands. However, we acknowledge that the Merowe reservoir will still receive an annual flood inflow due to inflows below the GERD that may require the pool elevation to be lowered somewhat prior to receiving this inflow. Our assumption provides a conservative estimate when evaluating the impacts of evaporation on Egypt.

The assumptions for the adaptation of Sudanese reservoirs are considered realistic to maximize the benefits and minimize the risks to Sudan, and therefore the analysis holds them constant throughout this study. This allows the methodology of seeking a negotiated coordination to be demonstrated between the key reservoirs of HAD and the GERD but assumes that Sudan would agree to any negotiated arrangement between Ethiopia and Egypt.

Initial conditions for all reservoirs are assumed to be full with the exception of the HAD, which is assumed to begin with an elevation of 175 m based on the annual flood limitation level. Travel times between the GERD and the HAD are approximately 15 to 25 days, which are included and calibrated as flow‐dependent lags throughout the system.

### 4.2 Problem Formulations

In addition to the predevelopment case, postdevelopment noncooperative and cooperative formulations are considered that represent steps and sequential iterations of the process shown in Figure 2. In all formulations, Ethiopia seeks to maximize average annual energy generation while also maximizing the reliability of the target power generated from the GERD. Sudan's objectives are to maximize average annual energy production and minimize the risk of shortages to water users. Egypt's objectives are also to maximize average annual energy generation and minimize the risk of shortages to water users, as well as minimize the risk of the HAD reaching the minimum operation level (MOL) of 147 m where severe and highly disruptive shortages could occur. Minimizing system losses including releases in excess of downstream demands and spills into the Toshka spillway is also considered as an objective.

A *predevelopment formulation (No GERD)* considers a scenario before the GERD is operational or does not exist in future scenarios. Current operations for Sudanese and Egyptian reservoirs are assumed to continue into the future. This formulation provides the basis for estimating the current risks of shortages in Sudan and Egypt.

A *noncooperative formulation (No Coop)* assumes that Ethiopia acts independently to maximize hydropower generation. Thus, in this scenario the downstream nations receive no information on the intended operational rules and therefore cannot predict how the GERD would be operated.

Assuming the objective of the GERD is to provide a power baseload to Ethiopia and adjacent countries with power purchase agreements (Degefu et al., 2015; DoP, 2015), the primary operation is to select a turbine release (*R*) that seeks to generate a specified target hydropower generation rate subject to the resulting elevations bounded by flood control elevations and MOLs:

$$\begin{array}{l} R=\min\left(\max\left(R_{\mathrm{HP}},R_{\mathrm{FC}}\right),R_{A}\right)\\ R_{\mathrm{HP}}=\frac{P_{T}}{H_{\mathrm{net}}\times \rho \times g\times \gamma }\\ \gamma =f\left(H_{\mathrm{Net}}\right) \end{array}$$

In these equations, *R* is the monthly release from the GERD; *R*_HP_ is the turbine release required to meet the target hydropower objective *P*_T_; *R*_FC_ is the release that does not allow the maximum flood elevation to be exceeded; *R*_A_ is the maximum release possible without falling below the minimum power pool elevation of 590 m; *H*_net_ is the net head; *ρ* is the density of water; *g* is the acceleration of gravity; and *γ* is the power plant efficiency. In this formulation, *P*_T_ is selected to maximize energy generation while also evaluating the reliability of power production at the selected power projection level. This assumed operation does not speculate whether changes to releases would occur to avoid these upper and lower bounds of the reservoir, but this information can be easily incorporated if available. This formulation is simply evaluated over a range of *R*_HP_ values to determine a rational choice for GERD operations and examining the downstream impacts.

In this formulation, downstream countries have no knowledge of upstream operations; therefore, adaptation measures are not presumed to occur. We recognize that a formulation without downstream adaptation reveals a *worst case* for each these countries but reveals the impacts of nonadaptation to the new flow paradigm on the Nile. As of October 2018, Sudan has recognized that they will need to quickly adapt to the new situation, but plans for Egypt to adjust the operations of the HAD have not yet been publically revealed.

A *basic cooperative formulation (Basic Coop)* is based on a minimum agreed annual release (AAR) volume from the GERD that is distributed evenly over the year and adaptations of the HAD operation to minimize risk based on this expected AAR. These adaptations include modifying current drought management policies by exploring different percentage reductions to HAD releases when the storage falls below 30, 35, 40, 45, and 50 BCM and reducing HAD releases under all conditions (i.e., above 50 BCM). At each of these storage zones, a range of reductions to releases are tested (0% to 15%), with nondecreasing reductions as storage levels fall. Adaptations to the HAD flood management policy is also explored by considering a range of maximum elevations (175 to 180 m) to be reached by 1 August to provide flood storage space.

This formulation assumes the Sennar, Roseries, and Merowe dams will maintain a maximum elevation whenever possible after direct diversions are made (Sennar), releases to meet downstream Sudanese demands are met (Roseries and Sennar), and releases for target hydropower are made (Merowe). Regular spills from all Sudanese reservoirs occur due to their small storage volume relative to average annual flows. This simple assumption might not be practical due to sediment management concerns; however, it provides a conservative estimate of evaporation losses.

The releases from the GERD seek to achieve target power generation, subject to an AAR that the countries may negotiate.

$$R_{t}=\min\left(\max\left(R_{\mathrm{HP}},\frac{\max\left(\left(\mathrm{AAR}-\sum_{T1}^{t-1}R_{i}\right),0\right)}{T2-t+1},R_{\mathrm{FC}}\right),R_{A}\right)$$

*T*1 is the starting month of the water year, and *T*2 is the final month of the water year. The target hydropower is assumed to remain constant, and the equation above distributes any volumes of AAR in excess of that which is required for power generation evenly throughout the water year. In this formulation, all downstream parties are assumed to have knowledge of an expected and regular release and can plan their operations accordingly. An AAR must be feasible under all hydrologic scenarios; therefore, any agreements that would deplete the GERD below a minimum power elevation (590 m) would not be viable due to insufficient water available to meet such an agreement. The drought management and flood control assumptions for the HAD are adapted to minimize risks of shortages and unnecessary spills in Egypt, and the Sudanese reservoirs downstream of the dam are maintained at the highest level possible.

A *continuous cooperative formulation (Cont Coop)* builds upon the Basic Coop formulation by dynamically protecting downstream users in the case of severe drought. This formulation combines the AAR and adaptations to the flood and drought operations of the HAD with a safeguard release (SR) from the GERD, which is triggered if the HAD pool elevation is predicted to go below an elevation threshold. A range of HAD trigger levels for the SR is tested in this formulation, ranging from the minimum HAD operation level of 147 m to a higher trigger level of 175 m. Variations of the percentage reductions for drought operations are simultaneously tested with varying SR trigger levels, allowing combinations of policies that impact Egypt to varying degrees. This includes an upper SR trigger level and low drought percentages reductions, resulting in fewer drought management restrictions on Egyptian water use at the cost of more frequent SR releases from the GERD. Conversely, this formulation also considers combinations of lower SR trigger levels and higher drought percentage reductions that would invoke more drought management restrictions on Egyptian water use while retaining water in the GERD. All possibilities between these extremes are evaluated within this formulation. The continuous cooperation formulation requires forecasting of inflows into Lake Nassar and assumes that all the information that is necessary to implement the formulation is shared among the countries. Furthermore, the SR water from the GERD to protect the elevation of the HAD is allowed to pass through the three intervening Sudanese reservoirs (Roseries, Sennar, and Merowe) by increasing their releases within the physical limitation of their outlet works; thus, Sudan would need to be party to such an agreement between Ethiopia and Egypt.

The Basic Coop and Cont Coop scenarios are evaluated using the MOEA search method. In the most complex cooperative formulation, the basin management seeks to optimize a vector of eight objectives *F*(*x*) using a vector of decision of variables *x*:

$$\begin{array}{l} F\left(x\right)=\left(f_{\text{EthEn}},f_{\mathrm{Eth}90\%\mathrm{Pr}},f_{\text{EgyEn}},f_{\text{SudEn}},f_{\text{SudShort}},f_{\text{EgyShort}},f_{\text{HADMOL}}\;f_{\text{EgyLoss}}\right)\\ x=\left(P_{T},\mathrm{AAR},F_{\mathrm{HAD}},D{\left(Y\right)}_{\mathrm{HAD}},{\mathrm{SG}}_{\mathrm{HAD}}\right)\\ Y=\left\{35,\;40,\;45,\;50,>50\right\} \end{array}$$

where *f*_EthEn_ is the average annual energy generated by the GERD, *f*_Eth90%Pr_ is the 90th percentile of monthly power generated by the GERD, *f*_EgyEn_ is the average annual energy generated by the HAD, *f*_SudEn_ is the total average annual energy generated by all the Sudanese hydropower dams in the eastern Nile Basin (Roseries, Sennar, Merowe, Khashm El Girba, and Jebel Aulia), *f*_SudShort_ is the average annual shortages to all Sudanese water users in the eastern Nile Basin, *f*_EgyShort_ is the average annual shortages to the combined demands downstream of the HAD and from the Toshka pumping project, *f*_HADMOL_ is the frequency of the HAD reaching the MOL of 147 m, and *f*_EgyLoss_ is the combination of the average annual spills to the Toshka canal and releases in excess of the Egyptian demands downstream of the HAD. The results of each objective are averaged across multiple hydrologic scenarios, thereby presuming that the distribution of inputs and outcomes is relatively normal. Although the search for decision variables is aimed at improving the outcome under average conditions, objectives from individual extreme conditions may fall above or below these averages. This is acceptable, since none of the objectives are assumed to be achieved in all conditions, particularly given the extreme variability of the Nile.

The decision variables *x* include *P*_T_, which is the target hydropower generation from the GERD; AAR is the agreed annual release from the GERD; *F*_HAD_ is the flood space elevation to be achieved by 1 August, *D*(*Y*)_HAD_ is the percentage reduction in deliveries from the HAD to Egyptian water users at reservoir storage *Y*, and *SG*_HAD_ is the HAD elevation triggering SR from the GERD. The *D*(*Y*)_HAD_ is equivalent to the current HAD drought management plan described earlier, but the configuration allows reductions to be applied when the reservoir is above 50 BCM (>50) and when it falls below the 50, 45, 40, and 35 BCM storage values. Shortages triggered at 50 BCM (i.e., below the current start of drought reductions at 60 BCM) was discovered to achieve similar results as the status quo, allowing for reductions in the search space of the MOEA algorithm.

Applying the ENRM with the MOEA, cooperation arrangements are explored by iteratively sampling combinations of management decisions outlined in Table 3 and evaluating the performance of the objectives outlined in Table 2. For both the Basic Coop and Cont Coop scenarios, Borg was run with default parameters (Hadka & Reed, 2013) except for assigning a search population of 50 and epsilon values of 0.01% for *f*_HADMOL_ and 1 for all other objectives. Search ranges and increments were selected based on existing reservoir characteristics, preliminary tests, and previous knowledge of the system dynamics. The algorithm was run for an average of 6,000 iterations, that is, exchanges between the operation of the hydropolicy model and recommended values from the Borg MOEA.

**Table 2:** Problem Objectives by Country

Ethiopia	Sudan	Egypt
Maximize energy *E* [*f*_EthEn_]_100_	Maximize energy *E* [*f*_SudEn_]_100_	Maximize energy *E* [*f*_EgyEn_]_100_
Maximize 90% power reliability *E* [*f*_Eth90%Pr_]_100_	Minimize shortages *E* [*f*_SudShort_]_100_	Minimize shortages *E* [*f*_EgyShort_]_100_
		Minimize problem of reaching MOL *E* [*f*_HADMOL_]_100_
		Minimize spills from HAD (Toshka + downstream) *E* [*f*_EgyLoss_]_100_

*Note*. *E*[…]_100_ denotes the expected value across 100 traces. MOL = minimum operation level; HAD = High Aswan Dam.

**Table 3:** Decision Variables Used in Each Formulation, Along With Borg Search Parameters

Decision Variable	Notation	No coop	Basic coop	Continuous cooperation	Borg search range	Borg search increment (*ε* value)
GERD target power (MW)	*P*_T_	X	X	X	800–1,800	50
GERD agreed annual release (BCM)	AAR		X	X	0–50	1
HAD drought reductions (%)						
Storage <35 BCM	*D*(35)_HAD_		X	X	0–15%	1%
Storage <40 BCM	*D*(40)_HAD_		X	X	0–15%	1%
Storage <45 BCM	*D*(45)_HAD_		X	X	0–15%	1%
Storage <50 BCM	*D*(50)_HAD_		X	X	0–15%	1%
Storage >50 BCM	*D*(>50)_HAD_		X	X	0–15%	1%
HAD flood space elevation (m)	*F*_HAD_		X	X	175–180	1
GERD‐HAD safeguard release elevation (m)	SG_HAD_			X	147–175	1

*Note*. GERD = Grand Ethiopian Renaissance Dam; HAD = High Aswan Dam; AAR = agreed annual release; BCM = billion cubic meters.

## 5 Results

### 5.1 First Phase: Approximating Stakeholder Criteria

In the first phase of the analysis, the perspectives of each stakeholder are identified and quantified. We assume that the preferred operation of Egypt and Sudan would be based on the condition before the GERD is constructed. The perspective of Ethiopia would be based on the assumption that the GERD is used for the single purpose of hydropower generation. As a result, stakeholder criteria are derived from both the predevelopment formulation and the noncooperative formulation.

#### 5.1.1 Predevelopment Formulation

**Figure 4:**
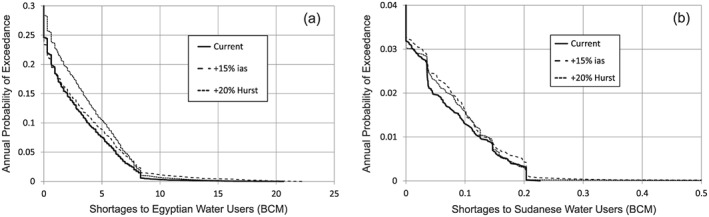
Annual probability of exceedance of shortages to (a) Egyptian and (b) Sudanese water users under current conditions, 15% increase in interannual standard deviation, and 20% increase in the Hurst coefficient.

According to international law, the downstream users, Egypt and Sudan, would expect that the GERD should not cause any *significant harm* (Salman, 2016), which is interpreted as avoiding any significant changes to current consumptive uses or energy production. Using the predevelopment formulation, Figure 4 demonstrates the distribution of annual shortages to downstream water users, where shortages are defined relative to the expected uses of 55.5 BCM for Egypt and 18.5 BCM for Sudan. Results are shown for current hydrologic conditions, as well as climate perturbed conditions: +15% ias and +20% Hurst. Under baseline hydrologic conditions, average annual shortages of 0.871 and 0.003 BCM are expected for Egyptian and Sudanese users, respectively. Maximum annual shortage volumes of 20.6 and 0.227 BCM for Egypt and Sudan, respectively, are currently possible but are only associated with the rarest droughts in our stochastic flow simulations. These shortages can be the result of current operational policies (i.e., drought management restrictions) or physical limitations of the amount of water available. The ability of selected strategies to mitigate risks of climate‐perturbed conditions is shown in subsequent stages of the analysis but provided here for clarity.

The predevelopment formulation is also used to derive the current condition average annual energy generation of 6.52 and 9.42 TWh derived from the all Egyptian and Sudanese dams, respectively.

Using this formulation, we propose succinct definitions for the requirements of the GERD to avoid causing significant downstream harm. For Egypt, we include the following three components: (1) the GERD would not cause the average annual shortages to Egypt to exceed the value expected without the presence of the GERD (0.817 BCM/year), (2) the probability of the HAD reaching the minimum operating level of 147 m would not exceed the risk that exists without the presence of the GERD (currently 0.14%), and (3) the average annual energy generation from the HAD would not be less than the value without the GERD (6.52 TWh/year).

The criteria for Sudan would follow a similar logic. To avoid significant harm, (1) the GERD would not cause the average annual shortages to exceed the expected value without the presence of the GERD (0.003 BCM/year) and (2) the average annual energy generation in Sudan would not be less than the value without the GERD (9.42 TWh/year).

#### 5.1.2 Noncooperative Formulation

**Figure 5:**
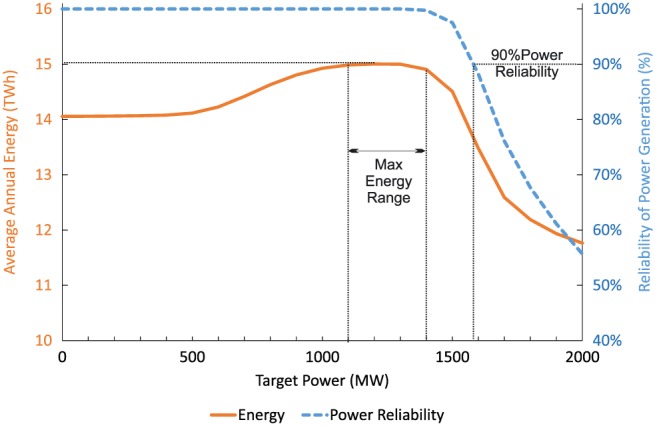
Energy production and reliability of power generated by the GERD when operated over a range of targeted hydropower production levels (*P*_T_). GERD = Grand Ethiopian Renaissance Dam.

As mentioned above, the results of the noncooperative formulation are used to determine the hydropower generation potential of the GERD, which represents the preferred position of Ethiopia. A range of target power generation levels was examined to determine average annual energy generation that the GERD could deliver under current hydrologic conditions (i.e., based on 1900–2002 flows) while noting reliability of meeting the target power specified. Figure 5 demonstrates that the GERD can provide up to 15 TWh/year on average, with 100% reliable power generation when producing between 1,100 and 1,400 MW. This range represents a rational choice for Ethiopia's management of the GERD, with the upper end maximizing both energy and constant power production (MaxEnergy). However, the reliability decreases rapidly if the dam is operated above 1,400 MW. A reliability of 90% is often used for firm energy generation (Ramachandra et al., 2000), which can be achieved with a target power of approximately 1,600 MW. At this power generation rate (90%Power), only 13.7 TWh of average annual energy can be produced. Both generation levels of 15 TWh/year and 1,600 MW were considered as potential starting negotiation positions for Ethiopia. Anything less on either metric can be considered a cost to Ethiopia. The area between 1,100 and 1,600 MW represents the likely range of normal operation of the GERD (Table 3).

The initial criteria for each stakeholder are summarized in Table 4, which are then passed into the second phase of the framework in Figure 2. For Ethiopia, the two rational operations are to either maximize the energy generation (MaxEnergy) or to maximize the power generation with 90% reliability (90%Power). The criteria for Sudan and Egypt are based on the predevelopment formulation and thus without the GERD.

**Table 4:** The Initial Assumed Acceptable (No Harm) Positions for Each Stakeholder With Current, +15% ias, and +20% Hurst Hydrologic Conditions

Criterion Name	Current	+15% ias	+20% Hurst
Ethiopia criteria (assumes with GERD)			
MaxEnergy: Maximum average energy (TWh/year)	15.00	15.00	14.96
90%Power: Maximum 90% reliable power (MW)	1,581	1,579	1,572
Sudan criteria (assumes without GERD)			
Average energy (TWh/year)	9.42	9.39	9.41
Average shortage (BCM/year)	0.0030	0.0036	0.0033
Egypt criteria (assumes without GERD)			
Average energy (TWh/year)	6.52	6.53	6.32
Average shortage (BCM/year)	0.871	0.993	1.145
Probability of MOL	0.14%	0.48%	0.27%

*Note*. ias = interannual standard; GERD = Grand Ethiopian Renaissance Dam; BCM = billion cubic meters; MOL = minimum operation level.

Table 4 also demonstrates how the initial positions would change under given scenarios of alterations to hydrologic persistence. While not explicitly used until the second phase of the process shown in Figure 2, information is presented here to understand the sensitivity of the initial positions to these uncertainties. The only significant change that can be seen is an increase in the average shortages to Egypt with increased variability and persistence. This demonstrates an increase of risk to Egypt regardless of the presence of the GERD and suggests that the GERD might help to mitigate this risk.

#### 5.1.3 Implications of Noncooperation

To quantify the risks of noncooperation among the countries with respect to the GERD operations, we evaluate the MaxEnergy and 90%Power alternatives described above and initially assume that the downstream reservoirs are not able to adapt to the GERD due to a lack of information. While this scenario is quite unlikely, it estimates the implications of not reaching an agreement regarding GERD operations and reveals the implications of not adapting.

**Figure 6:**
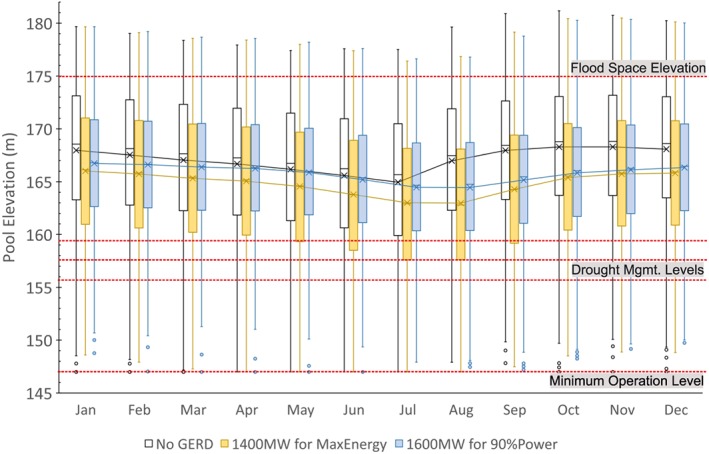
Monthly HAD pool elevations with two GERD operations and no downstream adaptations for current hydrologic conditions. HAD = High Aswan Dam; GERD = Grand Ethiopian Renaissance Dam.

The combined effects of changed timing of flows, increased evaporation losses from the GERD, and decreases in evaporation losses from Lake Nasser are shown in Figure 6. The average elevations resulting from both the MaxEnergy and 90%Power operations fall below the no‐GERD operations, resulting in a 0.30 and 0.14 TWh decrease in average annual energy generated from the HAD, respectively. The relatively steady releases from the GERD result in less frequent occurrences of the HAD reaching high elevations, suggesting the current flood management policy could be adapted to decrease proactive releases and thus conserve water, but at an elevated risk of emergency releases to manage unexpected large inflows. The probability of the HAD reaching low elevations that can trigger drought management operations differs between the two GERD management policies. The MaxEnergy policy makes comparatively lower hydropower releases from the GERD and therefore increases the probability of the HAD falling to lower elevations. The higher monthly GERD power output in the 90%Power policy results in steadier flows between the reservoirs and a more compact distribution of HAD elevations compared to the pre‐GERD behavior during the critical low flow months, resulting in fewer reductions to Egyptian water users. Without guarantees of how the GERD will be operated, however, Egypt is unable to adapt their shortage management strategies to benefit from the GERD.

**Figure 7:**
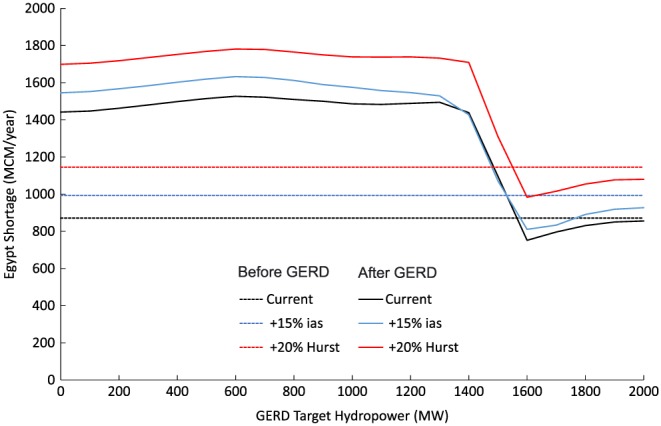
Annual shortages to Egypt as a function of GERD target power releases for noncooperation policy under current, +15% ias, and +20% Hurst hydrologic conditions. Reference lines before GERD are shown for comparison. GERD = Grand Ethiopian Renaissance Dam.

Figure 7 expands on this analysis by demonstrating the change to shortages for Egyptian water users assuming no adaptation of the HAD operations. The flat lines are shortages prior to the GERD provided for reference. The step change between 1,400 and 1,600 MW aligns with the switch from MaxEnergy to 90%Power (Figure 5). In this transition zone, the costs and benefits to Egypt are the most sensitive and demonstrate where negotiations may be the most effective.

We also note that without adaptation of the Roseries and Sennar reservoirs to the operations of the GERD, average shortages to Sudanese water users increases by 200% to 1,000%, demonstrating a clear need for adaptation (results not shown graphically). If releases from the GERD are known in advance, elevating the Sudanese reservoirs throughout the year and making releases only for irrigation and power generation is a logical adaptation response and the planned operation of the Sudanese government (Basheer et al., 2018). This assumption is used through this analysis as well.

### 5.2 Second Phase: Seeking Cooperation

#### 5.2.1 Basic Cooperation Management Solutions

**Figure 8:**
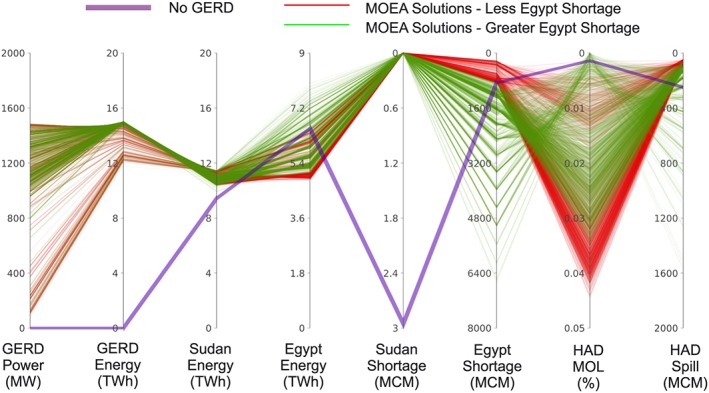
Parallel plots of multiple objectives with solutions discovered under basic cooperation. GERD = Grand Ethiopian Renaissance Dam; MOEA = multiobjective evolutionary algorithm; HAD = High Aswan Dam; MOL = minimum operation level.

The results of eight objectives in Table 2 can be visualized using parallel plots (Inselberg, 1985) where each vertical axis represents an objective and each segmented line represents the performance of a particular management solution (Figure 8). Figure 8 shows only the nondominated solutions, using a convention of the upward direction on each axis to be desirable, and diagonal lines demonstrate trade‐offs. The condition prior to the GERD is shown by the thick blue line (No GERD), and the red and green lines demonstrate solutions that decrease and increase the shortages to Egypt, respectively.

In this formulation, several solutions were identified that can lower the shortages to Egypt by decreasing the drought reduction tiers to lower elevations relative to those currently used, but no solutions were identified that both reduce the shortages to Egypt and reduce the probability of the HAD reaching the MOL. Energy gains for Sudanese reservoirs were achieved in all solutions, and changes to energy generation for Egypt generally mirror the shortages to Egypt, as both are driven by the effects of pool elevations.

**Figure 9:**
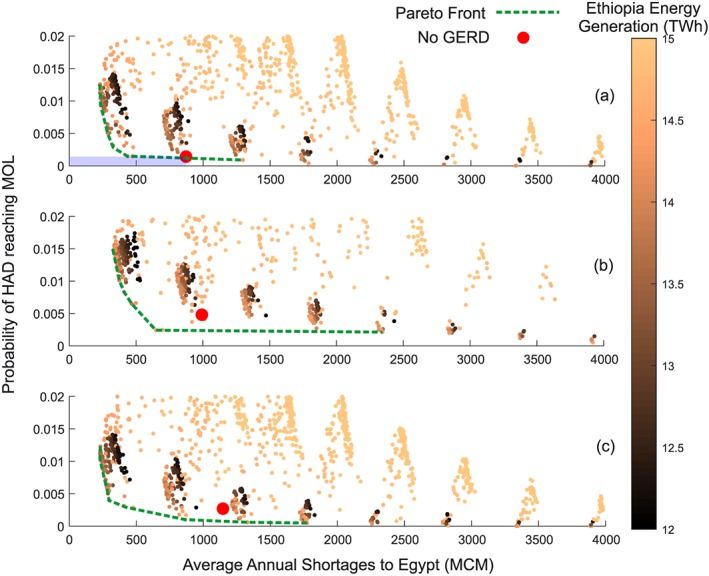
Primary risks to Egyptian water supply with management solutions discovered under basic cooperation. Figure 9a represents solutions under current hydrologic conditions, Figure 9b represents solutions under the +15% ias conditions, and Figure 9c represents solutions under current +20% Hurst conditions. The shaded area in 9a indicates that definitive improvements could not be identified under current conditions with respect to both axes variables. GERD = Grand Ethiopian Renaissance Dam; HAD = High Aswan Dam.

Figure 9 demonstrates a more in‐depth examination of the two critical dimensions for Egypt including the average shortages to Egyptian water users and the probability of the HAD reaching the MOL. Each point represents a nondominated solution, and the colors of the points represent the average annual energy generation by the GERD. The Pareto front of the two axes variables is shown on the left side (in green). The plots include the solutions under current (Figure 9a), the +15% ias (Figure 9b), and the +20% Hurst (Figure 9c) hydrologic conditions, along with the risks that exist without the GERD (red dot). Figure 9a demonstrates the risks under probable conditions, while Figures 9b and 9c demonstrate how the plausible climate induced hydrologic changes increases the risks to Egypt without the presence of the GERD and how the GERD can help to mitigate these risks.

As noted in the shaded area in Figure 9a, none of the discovered solutions is better with respect to both axes based on the current hydrologic conditions. However, reductions to the average annual shortages to Egypt are clearly possible with only minor increases in the risk of the HAD reaching the MOL. Given the various uncertainties in the model, though, a basic level of cooperation may indeed be sufficient to provide reductions of risks for Egypt and could be examined further by Egypt for acceptability. However, since this formulation cannot assure that our definition of no significant harm can be achieved under current conditions, and this paper aims to demonstrate the iterative nature of the method outlined in Figure 2, we reformulate the model to describe a new form of cooperation that may yield better outcomes and be more acceptable to the DMs involved in the negotiations.

#### 5.2.2 Continuous Cooperation Alternatives

**Figure 10:**
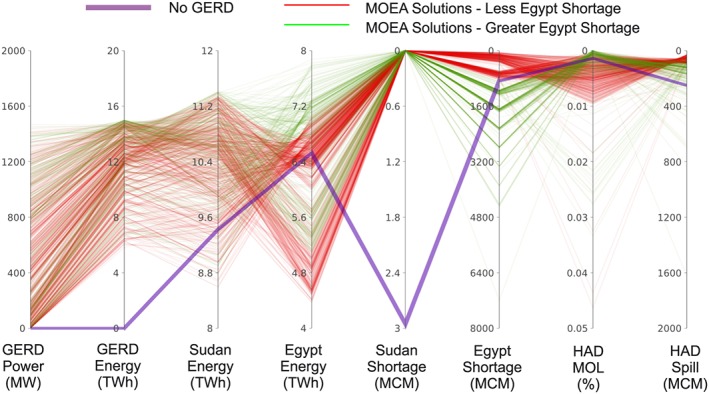
Parallel plots of multiple objectives with solutions discovered under continuous cooperation. GERD = Grand Ethiopian Renaissance Dam; MOEA = multiobjective evolutionary algorithm; HAD = High Aswan Dam; MOL = minimum operation level.

Providing a SR from the GERD if the HAD reaches a critically low pool elevation would further mitigate risks to Egypt. It also requires a closer form of cooperation as it implies adaptation of operations on the basis that information by all three countries is shared to achieve mutually acceptable outcomes. Figure 10 demonstrates performance in the eight objectives optimized under the continuous cooperation formulation. In this arrangement, several solutions can be identified that meet both the average annual shortage criterion for Egypt and the criterion of not exceeding the current probability of reaching the MOL. A nontrivial number of solutions also meets the criterion of not having significant impacts to the energy generation to Egypt. However, these coincide with both high levels of drought management reductions and hence high shortages to Egyptian water users, and solutions that specify high SR elevations, which lowers the reliability of power generation from the GERD.

**Figure 11:**
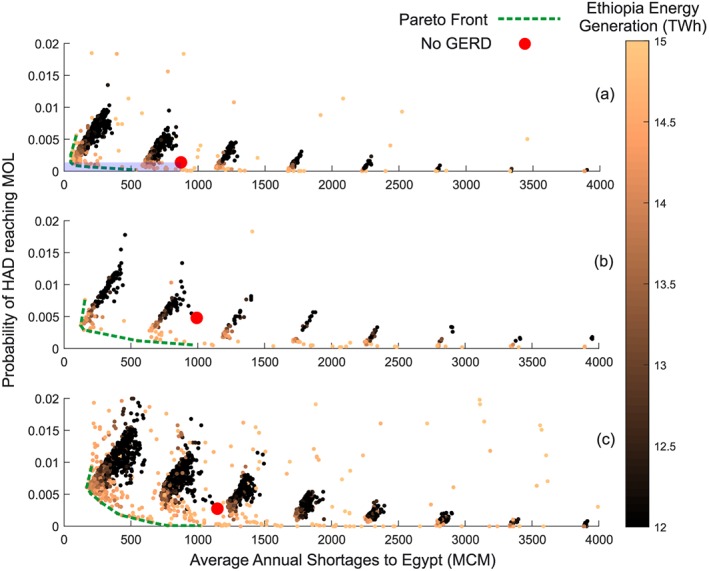
Primary risks to Egyptian water supply with management solutions discovered under continuous cooperation. Figure 11a represents solutions under current hydrologic conditions, Figure 11b represents solutions under the +15% ias conditions, and Figure 11c represents solutions under current +20% Hurst conditions. The shaded area in 11a indicates that many improvements could be identified under current conditions with respect to both axes variables. GERD = Grand Ethiopian Renaissance Dam; HAD = High Aswan Dam.

Figure 11 demonstrates the two key objectives for Egypt, shortages to Egypt and risk of the HAD reaching the MOL, using solutions derived from the continuous cooperation formulation. By comparing Figure 9 with Figure 11, it can be seen that substantial improvements can be made with respect to these objectives while maintaining high Ethiopian energy generation. Figure 11a indeed shows that the two criteria of no significant harm can be met for these objectives. Furthermore, Figures 11b and 11c demonstrate how risks to Egypt increase in the +15% ias and +20% Hurst scenarios, and while very helpful to protect future downstream water reliability, even SRs cannot fully protect Egypt in increasingly extreme events. Because the highly cooperative method of a SR can mitigate risks under many conditions, further exploration of specific solutions is warranted.

**Figure 12:**
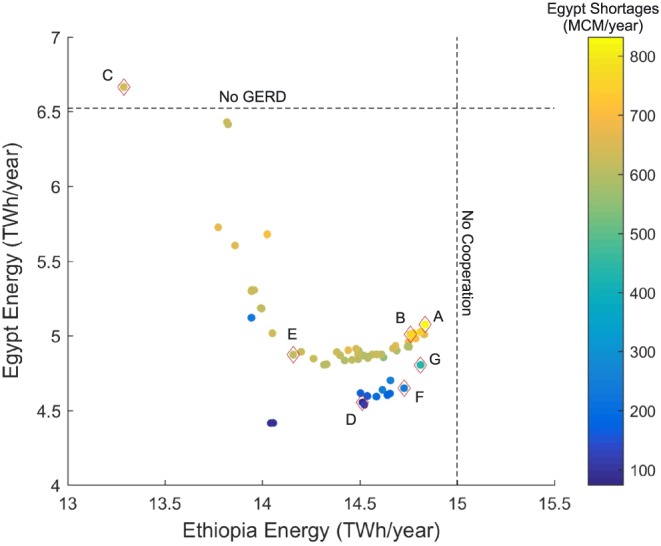
GERD‐HAD energy and trade‐offs with Egyptian shortages under continuous cooperation. GERD = Grand Ethiopian Renaissance Dam; HAD = High Aswan Dam.

A subset of solutions was selected that reduce the risks to Egyptian water supplies compared to the situation without the GERD under current conditions (i.e., solutions in the shaded area in Figure 11a). This set of solutions would meet our definition of no significant harm to downstream water users with respect to the criteria of no additional average annual shortages (less than 0.871 BCM/year) and no increased risk of reaching the MOL for the HAD (probability less than 0.14%). Using only these solutions, Figure 12 shows the trade‐off of costs in terms of the average annual energy generation for both Ethiopia and Egypt under current hydrologic conditions. The multimodal distribution is a result of the MOEA searching for solutions along the multiobjective Pareto fronts that seeks to minimize shortages to Egypt on one side and maximize Egyptian energy generation on the other. Solutions are shown relative to the No‐GERD and noncooperative management cases for Egypt and Ethiopia, respectively. This demonstrates that the SR can result in energy generation that is close to the noncooperative case if energy can be produced when the water is needed from the GERD.

### 5.3 Robustness of Solutions to Plausible Hydrologic Conditions

**Figure 13:**
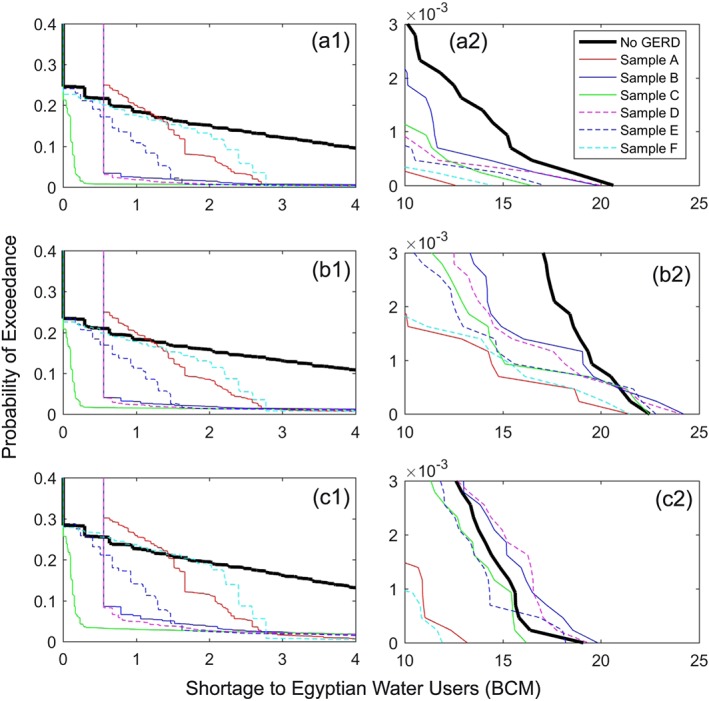
Annual probability of exceedance of shortages to Egyptian water users for sample with uncertain hydrologic persistence. Current conditions are shown in a1 and a2, +15% ias hydrologic scenario is shown in b1 and b2, and +20% Hurst hydrologic scenario is shown in c1 and c2. The left and right plots are different ends of the same exceedance curves.

A more specific analysis of the potential alternatives was performed considering increased variability and hydrologic persistence. A set of six *samples* was selected that met our definition of no significant harm to downstream water users, not including in terms of energy losses to Egypt, based on current hydrologic conditions (Figure 13). Having met this minimum criterion, the most helpful or least harmful of remaining dimensions could be examined based on principles of minimax theorem (von Neumann, 1928), which seeks to minimize the possible loss (e.g., minimize losses of hydropower) for a worst case scenario (i.e., Ethiopia agreeing to cause no harm to Egypt) or visually selected outcomes along the Pareto fronts. Sample A was chosen at the point of maximum Ethiopian energy generation, and sample B was selected at the point of maximum energy Egyptian generation. Sample C was selected to further minimize shortages to Egypt at the expense of other desirable outcomes. Samples D through F were selected to encompass the fronts and key inflection points of the shape shown in Figure 12. Using probable hydrologic conditions for this initial selection avoids potential complications of multistakeholder disagreements over which plausible climate perturbations are necessary to consider; however, the selections are then examined using these plausible conditions.

To evaluate robustness, each of the six samples was reevaluated under the current, +15% ias and +20% Hurst hydrologic ensembles. Figures 13a1 and 13a2 demonstrate the exceedance curves of annual shortages to Egypt using all these scenarios compared to the equivalent result for the No GERD case under current hydrologic conditions. Figures 13b1 and 13b2 demonstrate the exceedance curves of annual shortages to Egypt under the +15% ias scenario, and Figures 13c1 and 13c2 demonstrate the exceedance curves of annual shortages to Egypt under the +20% Hurst scenario. The plots on the left side (a) focus on the low magnitude shortages to Egypt, while the plots on the right side (b) focus on the infrequent high magnitude shortages.

All samples meet the criteria of no significant harm with respect to shortages to Egypt under current conditions. However, Figures 13a1 and 13a2 demonstrate that this is achieved in different ways across the scenarios. As can be seen in both these figures and in Table 5, samples A, B, and D assign a relatively aggressive drought management plan to the HAD, including a small reduction to Egypt deliveries under all HAD conditions. Conversely, Sample C almost eliminates the HAD drought management plan entirely and allows the HAD to decrease to lower levels, with the guarantee that the SR backup will protect the HAD under relatively low conditions, hence forgoing HAD energy generation. Sample B assigns a high SR trigger level (162 m), thereby protecting energy produced by the HAD and rarely needing to invoke the drought management plan.

**Table 5:** Potentially Viable Samples of High Cooperation Management Alternatives

Decisions	No GERD	Sample A	Sample B	Sample C	Sample D	Sample E	Sample F
		High GERD energy	High Egypt energy	Low Egypt short	Balance 1	Balance 2	Balance 3
Target power (MW)	—	1,039	855	836	1,107	1,287	951
AAR (BCM)	—	7	13	9	14	14	18
HAD SR elevation (m)	—	152	162	152	158	151	150
HAD flood elevation (m)	—	179	177	175	179	179	178
Egypt drought reductions at HAD storage levels							
>60 BCM	0%	1%	1%	0%	1%	0%	0%
<60 BCM	5%	1%	1%	0%	1%	0%	0%
<55 BCM	10%	1%	1%	0%	1%	0%	0%
<50 BCM	15%	1%	1%	0%	1%	0%	0%
<45 BCM	15%	3%	5%	0%	3%	0%	0%
<40 BCM	15%	13%	10%	1%	8%	4%	5%
<35 BCM	15%	13%	13%	1%	10%	10%	12%
*Objectives (average annual values)*							
Egypt short (BCM)	0.871	0.832	0.637	0.074	0.616	0.251	0.433
Sudan short (BCM)	0.003	0	0	0	0	0	0
GERD energy (TWh)	—	14.8	13.3	14.5	14.2	14.7	14.8
Sudan energy (TWh)	9.4	10.5	10.0	10.1	10.6	10.9	10.3
Egypt energy (TWh)	6.5	5.1	6.7	4.6	4.9	4.6	4.8
Egypt spill (BCM)	0.250	0.091	0.085	0.077	0.087	0.064	0.065
HAD probability of MOL (%)	0.14%	0.03%	0.13%	0.12%	0.11%	0.10%	0.07%
GERD 90% Reliable Power (MW)	—	1,039	608	836	979	1,287	951

*Note*. ias = interannual standard; GERD = Grand Ethiopian Renaissance Dam; AAR = agreed annual release; BCM = billion cubic meters; HAD = High Aswan Dam; SR = safeguard release; MOL = minimum operation level.

Focusing on robustness to the hydrologically perturbed scenarios, all samples maintain resilience to low magnitude shortages relative to the no‐GERD case when the variability or persistence is increased (Figures 13b1 and 13c1), notwithstanding the proactive management of Samples A, B, and D mentioned earlier. With respect to the risk of large magnitude shortages, the primary factor to protect against increases to variability and persistence is again the elevation of the SR trigger. Samples A, C, and F, maintain a low SR trigger level, thereby maximizing the amount of water retained in the GERD until it is needed downstream. Moderate releases from the GERD are also selected that are below the 1,100‐ to 1,600‐MW range identified in Figure 5. Samples B and D tend to result in occasional higher instances of large shortages relative to the No GERD case due to the higher SR trigger and more frequent releases to back up the HAD, even if this water is not immediately needed and then subjected to greater evaporation losses.

## 6 Implications for Infrastructure Operation on the Blue Nile

Our results show that noncooperative operation of the GERD to maximize hydropower benefits for Ethiopia would not provide assurance against negative impacts for downstream users. If explicit assurances of expected releases from the GERD can be made, and knowledge about planned releases provided well in advance, the operations of the HAD could be adjusted to minimize the additional risks faced by Egypt. Existing drought management policies of the HAD can be adapted to minimize the need to reduce releases to downstream users. Such an adaptation would result in Lake Nasser maintaining a lower elevation and thus a decreased margin of safety for Egypt. This suggests that close cooperation with Ethiopia would be advantageous, if not necessary, to eliminate any additional risks to Egypt.

Our analysis also demonstrates that a target power generation and AAR are effectively redundant, and these two factors can be negotiated concurrently as either a guaranteed release or a guaranteed power generation from the GERD. While our analysis also demonstrates that existing flood management policies could be adapted to allow a smaller flood storage space, this may not be advisable for safety reasons in the occurrence of unexpected inflows.

Provision of SRs from the GERD if the pool elevation of the HAD is projected to fall to a prespecified safeguard level would enhance the security for Egyptian water supplies. Allowing the HAD to operate lower, with a guaranteed back‐up from the GERD, minimizes evaporation losses but requires a higher degree of trust and coordination. Simultaneously specifying the GERD release requirements, the drought management characteristics of the HAD, and the GERD‐HAD safeguard policy allows the GERD to become beneficial to Egypt's water supply.

While the energy benefits from the GERD are substantial for Ethiopia (around 15 TWh/year), the results imply a likely reduction in hydropower production in Egypt. Samples that were considered viable in terms of no significant additional risk to Egyptian water users resulted in a range of annual HAD hydropower losses from 2.1 TWh to a slight gain of 0.1 TWh, based primarily on how the HAD drought management plan could be adapted and the specification of a SR trigger elevation. For Ethiopia to operate the GERD in a way that would not cause significant harm in terms of increased risk to Egyptian water supplies, it would require an Ethiopian energy reduction of 0.2 to 1.70 TWh/year compared to the maximum 15 TWh/year. Furthermore, the GERD would need to be operated at or below the low end of its target power range (1,100 to 1,600 MW) for Egypt to realize their own agricultural and municipal benefits of the GERD withholding water.

Simply put, if no safeguard policy is in place, it is to the advantage of Egypt for the GERD to operate at a maximum power generation rate (Figure 6). However, if a safeguard policy is agreed upon, then conserving water behind the GERD and allowing the HAD to operate lower to minimize evaporation losses would be preferred by all, including water users in Egypt. If Ethiopia is able to generate electricity when additional releases are needed to meet the safeguard elevation of the HAD, then only minimal energy losses need to be incurred by Ethiopia to provide this water. However, if there is not an energy demand at the time of the needed release, then water used to meet these downstream needs must be bypassed around the turbines, incurring a loss for Ethiopia. This emphasizes the advantage of the GERD being connected to a power pool capable of absorbing the energy produced whenever the water is needed downstream.

Selecting a range of possible scenarios of hydrological persistence has helped to further analyze various cooperative strategies in order to identify combinations that are robust to possible changes in hydrologic variability and drought persistence. Three of the samples (A, B, and D) suggested that a proactive reduction in releases to Egypt could be used to avoid the risk of larger shortages while maximizing other objectives (GERD and HAD energy output), and three samples (C, E, and F) demonstrated that future shortages to Egypt can be rare if a SR policy could be established between the countries and water is made available when it is called upon. Two of these selected samples (C and E) demonstrated that shortages to Egypt could be reduced across 99% of the perturbed hydrologic conditions, demonstrating their robustness to the particular hydrologic changes. One of these (sample E) suggests a configuration in which the GERD would not only improve reliability of water to Egypt under climate‐altered conditions but also allow the GERD to generate 14.7 TWh of electricity annually and 1,290 MW with 100% reliability, which is within the range of expected production rates during unilateral operations (Figure 5). All of the samples effectively eliminated the risk of shortages to Sudan.

The focus of this study is to support a politically based decision‐making process with analytical tools; therefore, assumptions of water use are fixed through time at a condition we considered to be *full utilization* by the three countries. However, this assumption is not an endorsement of any particular water allocation, nor does it account for dynamically changing demands over time due to new developments or increased crop demands resulting from a warmer climate.

While the emphasis of this work is to provide methods and insights to better navigate the process of negotiations for the Nile and the introduction of the GERD, it also suggests a number of possible directions for future work. Adaptations of Sudanese reservoirs were assumed based on the simplified assumption that these reservoirs are operated to meet Sudanese needs; however, the method could also be expanded to search for alternative management strategies of these intermediate reservoirs. Such strategies could seek to meet the needs of Sudanese agriculture and hydropower development while also protecting Egypt in times of drought. Consideration of future energy and agricultural demand projections would extend the uncertainty analysis. A further consideration would be to incorporate exchanges of benefits among the three countries by means of trade and power interconnections. Given that some formulations of a negotiated settlement involve accepting hydropower production that is less than the theoretical optimum, this could be compensated for with power transmission or financial transfers.

Although extensive efforts were made to collect information on reservoir operations, there is no certainty that future operations will align with the assumptions used. In particular, we presume the GERD will release water primarily to meet baseload energy demands; however, the turbine capacity allows the dam to meet energy peaking demands. Alternatively, Ethiopia could choose to make seasonal releases with a way that is conducive to downstream human and ecosystem needs in Sudan. Furthermore, we assume that Sudan acts in a supportive way by not intercepting SRs from the GERD that are intended for the HAD. These variations do not simply demonstrate the assumptions made for this study but also suggest how the parties might choose to expand the number of variables to match the items under consideration in the negotiations. The management configurations between reservoirs are numerous, so we provide this analysis not as a set of definitive solutions but instead deliver the partially automated methodology in Figure 2 to seek mutual gains in an exploratory setting. The attraction of our approach is that it could be adapted to include any formulation that is arrived at by the actors during negotiation.

## 7 Conclusions

Water resource models are commonly used to address the challenges of multiobjective decision making and suggest idealized or more efficient reservoir operations to meet those objectives; however, the processes by which tools are used to support negotiations over transboundary river development is less systematic, in part because of the highly political circumstances that are involved. The situation of the Nile exemplifies this challenge with the GERD soon to be added to the existing water management infrastructure on the river. Regardless of significant evidence highlighting the benefits of basin‐wide cooperation between Ethiopia, Sudan, and Egypt, the practical challenges of reaching agreements among these affected countries over the coordination of this infrastructure has not been adequately addressed with existing analytical tools and studies.

We have presented an approach that is perhaps less ambitious but more pragmatic than reconfiguring the operation of the entire river and instead demonstrate how a flexible hydropolicy model can be coupled with an MOEA to explore potential solutions that incrementally adapt the current reservoir operations. This exploration starts with the preferred positions of the countries and then uses both a mathematical search algorithm and iterative formulations of how the countries can choose to cooperate. This provides a structured way that models can be used to provide input to the negotiation process yet allows flexibility for creative solutions to emerge. With respect to the situation on the Nile, the method demonstrates not only how the GERD can be added to the existing system but also how the current operations of the HAD can be adapted alongside specific and agreed operations of the GERD.

In this study, we also address the uncertainty that future climate conditions pose on a negotiation process by a pragmatic approach that first identifies possible solution based on probable hydrologic conditions then evaluates these solutions under a wider range of plausible future conditions. The approach avoids the need to predetermine precisely which uncertain future conditions an agreement must be designed for but still allows future variations to be examined to inform their relative importance in the negotiations. In the case of the Nile, we used variants in the interannual variability and persistence characteristics of the flow series as examples to test the robustness of potential negotiated coordination management solutions and demonstrated various arrangements that are robust to these futures. Both climate‐induced hydrologic perturbations demonstrated increasing risks to Egypt in the absence of the GERD, but cooperative solutions between the GERD and the HAD could be identified that effectively eliminated these increases in future risks. Relatively speaking, the scenario that increased the drought persistence posed a greater risk compared to scenario of increased in interannual variability, indicating a need for the countries to monitor and plan for both longer and more severe droughts.

Future research will explore a wider range of variants on flow conditions that are consistent with emerging knowledge of climatic changes in the Nile Basin. Climate‐driven changes to agricultural demands for water are also likely to exceed currently assumed allocations, which can be incorporated into the uncertainty structure of the problem formulations. Furthermore, the development of additional infrastructure on the Blue Nile and within the Equatorial Lakes will require continuous renegotiation of coordination mechanisms.

Rapid recent advances in the field of multiobjective decision making have mostly failed to have an impact on complex and politically charged transboundary negotiations. We hypothesize that is because DMs struggle to connect the solutions generated by multiobjective optimizations with the realities of the systems that they are operating. The process described in this paper engages explicitly with the political as well as the technical challenges of finding mutually agreeable management arrangements. It seeks to build incrementally on recognizable management arrangements (starting with the status quo) and is structured around future cooperation principles that are salient in the negotiation process. We hope that it will prove to be applicable to other challenging transboundary river management situations as well as contribute to a negotiated settlement on the Nile.
